# A systematic review of prevention strategies for type 2 diabetes in First Nations children and young people

**DOI:** 10.1111/ijpo.70009

**Published:** 2025-03-10

**Authors:** Marylin Carino, Jonathan Nguyen, Ru Hui New, Renae Kirkham, Louise Maple‐Brown, Shiree Mack, Diana MacKay, Angela Titmuss

**Affiliations:** ^1^ Wellbeing and Preventable Chronic Diseases Division, Menzies School of Health Research Charles Darwin University Darwin Northern Territory Australia; ^2^ Department of Paediatrics Division of Women, Children and Youth, Royal Darwin Hospital Darwin Northern Territory Australia; ^3^ Department of Endocrinology Royal Darwin Hospital Darwin Northern Territory Australia

**Keywords:** children, First Nations, paediatrics, systematic review, type 2 diabetes

## Abstract

**Introduction:**

There is a high prevalence of type 2 diabetes (T2D) in First Nations populations worldwide, increasingly at younger ages. This review aims to identify interventions for the prevention of T2D in First Nations children and young people aged 4–25 years.

**Methods:**

A systematic search of both published and unpublished literature until March 2024 was performed using 15 electronic databases, including MEDLINE, CINAHL, EMBASE, Scopus, Cochrane Library, ATSIHealth, OpenGrey and specific First Nations databases. Eligible studies included First Nations participants aged 4–25 years without T2D, exploring interventions to prevent T2D. Outcomes included knowledge of diabetes, anthropometry and physiology, diet and nutrition, physical activity, glycemic indicators and psychosocial indicators.

**Results:**

Fourteen pre–post exposure non‐controlled studies were included, evaluating nine programs. Programs were culturally adapted and primarily school‐based, focusing on individual‐level behaviour modification in nutrition and physical activity. Most studies assessing knowledge outcomes reported improvement. There were inconsistent findings regarding impacts on dietary intake and glycemia. One home‐based program achieved improvements across a range of outcomes, including body mass index, physical activity and psychosocial scores.

**Conclusion:**

Despite the increasing prevalence of T2D in First Nations children and young people, evidence of effective preventive strategies within these populations remains limited.

## 1 INTRODUCTION

First Nations children and young people have among the highest rates of youth type 2 diabetes (T2D) globally.^1^ Historically diagnosed in middle‐aged and older adults, the trajectory of youth‐onset T2D (defined here as a diagnosis of T2D before the age of 25 years) is concerning, being associated with a more rapid decline in pancreatic function, earlier development of complications, reduced quality of life, and increased mortality than later‐onset T2D, necessitating tailored interventions that address these differences.^2^, ^3^, ^4^, ^5^ First Nations children and young people are at a higher risk of developing T2D compared to their non‐indigenous counterparts,^6^, ^7^, ^8^ with established patterns of intergenerational transmission.^9^ Intergenerational risk may underlie shifting phenotypes of youth‐onset T2D compared to later‐onset T2D.^10^

There are likely complex factors underlying the disparity in diabetes prevalence between First Nations and non‐Indigenous youth, including social determinants of health, in‐utero exposure to maternal diabetes, obesity and metabolic dysfunction in pregnancy, rapid societal change, structural discrimination and food insecurity.^11^, ^12^, ^13^, ^14^ Additionally, First Nations populations have a shared history of colonization that has caused long‐lasting effects of systemic inequities, psychosocial stress and poorer health outcomes.^15^ Colonization has disrupted traditional ways of life for many communities, including food systems and physical activity patterns, leading to a high prevalence of risk factors for T2D.^15^

Preventing early onset T2D is critical, as optimal diabetes management may significantly decrease the risk of future complications.^10^ Comprehensive, community‐centred and culturally adapted approaches that recognize both historical and present‐day factors contributing to the diabetes epidemic among First Nations youth are necessary. Given the unique cultural, social and economic challenges facing First Nations children and young adults, it is crucial to examine the effectiveness of these approaches for diabetes prevention. Previous reviews have explored interventions to prevent T2D among First Nations adults, highlighting the potential of culturally adapted and participatory community‐based diabetes prevention interventions.^16^, ^17^, ^18^ These initiatives aim to reclaim traditional food systems, revitalize cultural practices, and empower individuals to make healthy lifestyle choices. However, none of these reviews have focused on children and young people. Evidence of effective interventions to prevent T2D in First Nations children and young people is needed in light of the differing phenotype and pathophysiology of youth‐onset T2D compared to adult‐onset T2D, and the distinct developmental stage of children and adolescents compared to adults. Young people may require better‐targeted lifestyle interventions that address both metabolic health and psychosocial factors influencing their health behaviours, suggesting the need for age‐specific tailored approaches compared to those used in adults. The purpose of this study is to systematically review the existing evidence of T2D prevention programs in First Nations children and youth, summarize their key outcomes, assess cultural and traditional components and explore key themes related to program design, implementation and engagement with First Nations communities. In this review, the term ‘First Nations’ will be used respectfully to refer to First Nations and Indigenous peoples globally.

## 2 METHODS

### 2.1 Study selection

This systematic review was designed and conducted in accordance with the Preferred Reporting Items for Systematic Reviews and Meta‐Analyses (PRISMA).^19^ The study protocol was prospectively registered (PROSPERO Registration Number: CRD42020208707). Studies were included if they assessed non‐pharmacological interventions aimed at preventing T2D in First Nations children and young people aged 4–25 years and not previously diagnosed with T2D; were culturally adapted; and were published in the English language. Studies needed to involve a comparison group and a total follow‐up duration of at least 12 months. This time period for follow‐up was chosen to assess the maintenance of any outcomes of the intervention. Studies that also examined non‐First Nations groups were eligible if data for First Nations participants were reported separately. There were no limitations on publication year. Conference abstracts, books and unpublished clinical trials were excluded.

### 2.2 Outcome measures

Outcomes of interest included diabetes knowledge, diet and nutrition, physical activity and fitness, anthropometry (body mass index [BMI], waist circumference, skinfold thickness, fat mass and adiposity), glycemic indicators (glycated haemoglobin [HbA1c], fasting glucose and insulin levels) and psychosocial indicators (quality of life [QoL] and depression).

### 2.3 Data sources and search strategy

A systematic search of both published and unpublished literature was conducted up to March 2024 using the following electronic databases: MEDLINE, CINAHL, PsycINFO, Joanna Briggs Institute, EMBASE, Scopus, Cochrane Library, ATSIHealth, FAMILY‐ATSIS, OpenGrey, ClinicalTrials.gov, Circumpolar Health Bibliographic Database, Informit Indigenous collection, Native Health Database and First Nations Studies Portal. The search strategy was customized for each database and included derivations of the terms: paediatric* or child* or young person or youth or adolescents or young adults or teen* AND First Nations or Native or Aboriginal AND type 2 diabetes or diabetes mellitus type 2 or diabetes 2 AND prevent* or intervention (see Appendix A). Reference lists from relevant review articles were also hand‐searched.

### 2.4 Data collection

**FIGURE 1:**
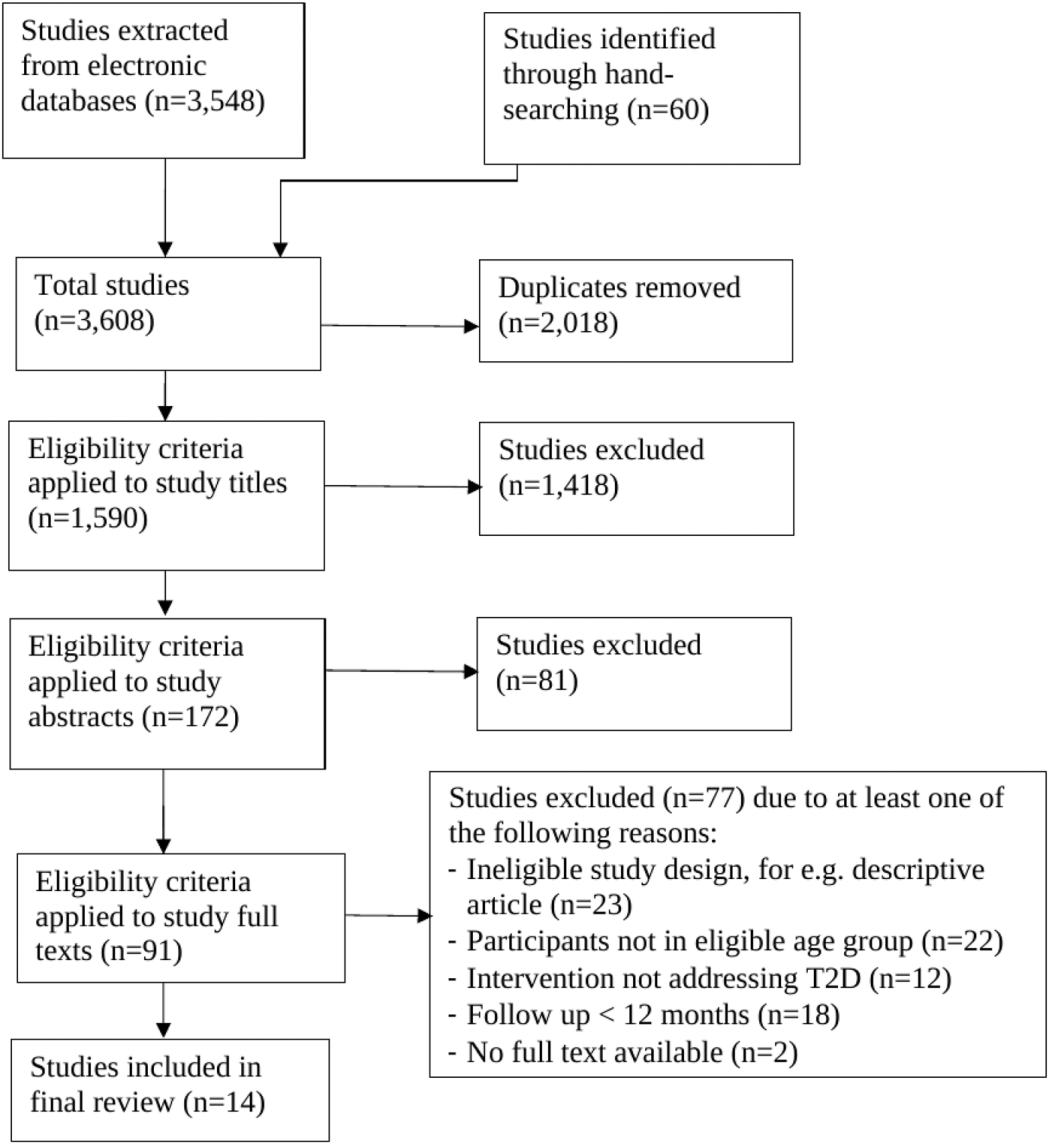
Flowchart of search process.

All search results were imported into EndNote, a software program that assists in the organization of literature searches. Duplicates were removed, followed by a review of the titles and abstracts of each study for eligibility criteria by authors M. C., J. N. and R. H. All studies selected for full‐text review were further screened independently for eligibility by the three authors using standardized screening tools, such as the Cochrane data collection form for randomized control trials (RCT) and non‐RCTs,^20^ and the Joanna Briggs Institute data extraction tool for qualitative research.^19^ Studies subject to discrepancies at each stage of eligibility screening were resolved through consensus between AT and DM. A summary of the search process is presented in Figure 1.

Data from the final included studies were extracted using the Cochrane data collection form and summarized in an Excel spreadsheet by M. C., J. N. and R. H. Data extracted included author, year, study design, participant demographics, intervention characteristics, culturally adapted components and outcomes. The studies involved varied considerably, measuring different outcomes, using different data collection methods and involving different populations and study durations. Due to the heterogeneity of the outcomes measured, meta‐analysis was not performed. Findings were reported in a narrative format, summarizing the results in text and tabulating the data rather than statistically pooling them together. This approach allows for a more nuanced understanding of the results while acknowledging the differences across studies. The risk of bias in each study was evaluated by M. C., J. N. and R. N. using the Cochrane risk of bias tool.

## 3 RESULTS

A flowchart of the search process is outlined in Figure 1. A total of 3608 studies were identified, with 1590 remaining following the removal of 2018 duplicates. A further 1499 studies were excluded after the title and abstract review, with 91 studies included for full‐text review. In the final review, 14 studies on 9 intervention programs were included, which are summarized in Table 1.

**TABLE 1:** Intervention and study characteristics.

Intervention program	Study author, year, country	Study design	Study participants	Intervention components	Examples of a traditional approach	Duration
Zuni high school diabetes prevention program (ZDPP)	Teufel 1998^21^ USA	Before–after study Cross‐sectional evaluation: 0,2 years	School years 9–12 Native American Indian Participants (*n*): 119/173	School‐based program incorporating integrated diabetes curriculum, development of school Teen Wellness Centre, summer break physical activities, school environmental component banning soft drinks.	Zuni Pueblo community collaborated with school district and university in developing program	4 years
	Ritenbaugh 2003^22^ USA	Before‐after study Cross‐sectional evaluation: 0,1.5,3 years	High school youth aged 16–18 years Native American Indian Participants (*n*): 70/64/65			
Many Rivers Diabetes Prevention Project (MRDPP)	Gwynn 2014^23^ Australia	Before–after study Cross‐sectional evaluation 0,4 years	School years 5‐8 Indigenous Australian Participants (*n*): 251/240	School‐based nutrition and physical activity programs. One‐off community events.	Traditional games and training with ‘bush tucker’ incorporated into healthy nutrition.	4 years
Kahnawake School Diabetes Prevention Project (KSDPP)	Jimenez 2003^24^ Canada	Before–after study Cross‐sectional evaluation 0,4 years	School years 4–6 Native North American Indian Participants (*n*): 156/146	School‐based health education curriculum including 10 45‐min duration lessons each year for every school grade, with topics including T2D, healthy nutrition and physical activity and fitness. Numerous school and community activities (such as a 2‐day walk physical activity, radio advertisements, promotional events), school environmental component banning junk foods.	Healthy nutrition lessons including traditional foods.	8 years
	Paradis 2005^25^ Canada	Before–after study Mixed: cross‐sectional evaluation (0,2,4, 5,8 years) and with non‐equivalent comparison group (0,2 years)	School years 1–6 Native North American Indian Intervention participants (*n*): 458/466/401/407/420 Comparison participants (*n*) 199/195			
	Adams 2005^26^ Canada	Before‐after study Cross sectional evaluation 0,4 years	School years 4‐6 Native North American Indian Participants (*n*): 150/145			4 years
The Sandy Lake Health and Diabetes Project: The Sandy Lake School‐Based Diabetes Curriculum (SLHDP)	Saksvig 2005^27^ Canada	Before–after study Longitudinal evaluation 0,1 years	School years 3–5 Native North American Indian Participants (*n*): 122	School‐based program including health education curriculum, family education (e.g., letters to parents), peer role‐modelling, healthy school meals, environmental component banning high‐fat foods.	Program developed with a local Oji‐Cree teacher with input from elders in the community involving storytelling lessons with Aboriginal characters.	1 year
The Sandy Lake Health and Diabetes Project: The Sandy Lake School‐Based Diabetes Curriculum Round 2 (SLHDP2)	Kakekagumick 2013^28^ Canada	Before–after study Cross‐sectional evaluation 0,0.5,1,1.5 years	School years 3–4 Native North American Indian Participants (*n*): 44/47/47/47	School‐based program as above with addition of greater emphasis on physical activity.	Program developed with a local Oji‐Cree teacher with input from elders in the community involving storytelling lessons with Aboriginal characters.	1–2 years
Good Start Program (GSP)	Mihrshahi 2017^29^ Australia	Before–after study Longitudinal evaluation 0,1 years	School children aged 6–19 years Maori and Pacific Islanders Participants (*n*): 375	School‐based program delivered by multicultural health workers including diaries monitoring healthy lifestyles, physical activities, nutrition education programs, training high school leaders.	Program employed multicultural health workers responsible for cultural tailoring and building community relationships.	1 year
Together on Diabetes (TOD)	Kenney 2016^30^ USA	Before–after study Longitudinal evaluation 0,0.25,0.5,1 years	Young persons aged 10–19 years Native American Indian Participants (*n*): 256	Bi‐weekly home‐based education by family health coaches (FHC) focusing on nutrition, physical activity and life skills. FHCs also provided social support to youth, facilitated community service referrals and hosted wellness events.	Family health coaches were paraprofessionals from the tribal community.	1 year
	Ducharme‐Smith 2021^31^ USA	Before–after study Longitudinal evaluation 0,1 year	Young persons aged 10–19 years Native American Indian Participants (*n*): 240			
	Chambers 2021^32^ USA	Before–after study Longitudinal evaluation 0,0.5,1 year	Young persons aged 10–19 years Native American Indian Participants (*n*): 256			
Youth Wellness Program (YWP)	Teufel‐Shone 2014^33^ USA	Before–after study Longitudinal evaluation 0,2 years	School years 3–8 Native American Indian Participants (*n*): 71	Twice‐weekly in‐school physical activity sessions aimed at improving strength and cardiovascular fitness.	A tribe‐university research team collaborated and ran program.	2 years
BRAID‐Kids project (BKP)	Toth 2012^34^ Canada	Before–after study Longitudinal evaluation 0,1 year	School years K4–9 Native North American Indian Participants (*n*): 22–72	School curriculum providing health education and promoting physical activity based on modified KDSPP/SLHDP.	‘Tradition‐based’ Cree Pride activities.	3 years

Abbreviations: *n*, number of participants; T2D, type 2 diabetes; USA, United States of America.

### 3.1 Study design and participant characteristics

All 14 studies included in this review were non‐controlled pre‐ and post‐exposure in design^21^, ^22^, ^23^, ^24^, ^25^, ^26^, ^27^, ^28^, ^29^, ^30^, ^31^, ^32^, ^33^, ^34^: 7 studies were repeated cross‐sectional evaluations,^21^, ^22^, ^23^, ^24^, ^25^, ^26^, ^28^ while 7 studies performed longitudinal evaluations.^27^, ^29^, ^30^, ^31^, ^32^, ^33^, ^34^ One study performed a mixed cross‐sectional analysis of its high school cohort population and a longitudinal analysis of specific high school participants with a non‐equivalent comparison community.^25^ This comparison population had its own diabetes prevention programme.

The 14 studies reported on 9 different intervention programs, with implementation ranging from 1994 to 2014, and are outlined in Tables 1 and 2. The Sandy Lake School‐Based Diabetes Curriculum program (SLHDP)^27^ was implemented and evaluated twice, with greater emphasis on physical activity evaluation in the second round of implementation (Sandy Lake School‐Based Diabetes Curriculum 2 program, SLHDP2).^28^ Two studies performed their evaluations before the completion of their respective programs: the Zuni Highschool Diabetes Prevention Program (ZDPP)^21^, ^22^ and the Kahnawake School Diabetes Prevention Project (KSDPP).^24^, ^25^, ^26^ The nine programs took place in Canada (*n* = 4), the United States (*n* = 3) and Australia (*n* = 2). The study populations all included First Nations school‐aged children, with participant ages ranging from 4 to 19 years.

**TABLE 2:** Study results.

Intervention program	Study author, year, country	Outcomes measured	Final follow‐up	Main results	Risk of Bias
Zuni high school diabetes prevention program (ZDPP)	Teufel 1998^21^ USA	Changes in BMI, pulse rate, dietary intake, blood glucose and insulin	2 years	‐ Downward trend in BMI (not significant), numerical data not available ‐ Decreased percentage of ‘high sugar content’ beverages consumed from 80% of beverages to <50% (*p* < 0.05) ‐ No change in dietary fibre intake ‐ Lower sitting pulse rates (*p* < 0.05, data not available) suggesting improved cardiovascular fitness. ‐ Decreased insulin levels at fasting and 30 mins postprandial (*p* = 0.005 for males, actual values not available) with increased glucose/insulin ratios suggesting decline in hyperinsulinemia prevalence.	Selection bias: Low Intervention/performance bias: N/A Detection bias: Low Attrition bias: Low Reporting bias: Low
	Ritenbaugh 2003^22^ USA	Changes in BMI, dietary intake, use of fitness centre, blood glucose and insulin	3 years	‐ No difference in BMI ‐ Increased daily mean use of wellness facility (24–115 students) ‐ Decreased consumption of sugar‐sweetened soft drinks at school (800 cans per week at baseline to 0 at follow‐up) ‐ Downward trend in median and 75th centile fasting/30‐min insulin levels. E.g., median regression coefficients for males were 72 vs. 113 for fasting, 586 vs. 1120 (both *p* < 0.001) for 30 min.	Selection Bias: Low Intervention/performance bias: N/A Detection bias: Low Attrition bias: Low Reporting bias: Low
Many Rivers Diabetes Prevention Project (MRDPP)	Gwynn 2014^23^ Australia	Changes in diabetes knowledge, weight and BMI, dietary intake, physical activity	4 years	‐ Increasing trend in proportion of boys correctly answering 90% of the diabetes knowledge questions (*p* = 0.11) ‐ More boys reported consuming less diet soft drinks (*p* = 0.0001) and girls less fruit juice (*p* = 0.0001). For e.g. girls consuming >2 cups/day decreased from 28% to 16%. However, reported intake of sugary drinks remained stable ‐ Stable results in most areas (other diet such as fruit intake, physical activity, weight)	Selection bias: High Intervention/performance bias: N/A Detection bias: Low Attrition bias: High Reporting bias: Low
Kahnawake School Diabetes Prevention Project (KSDPP)	Jimenez 2003^24^ Canada	Changes in dietary intake	4 years	‐ No differences in mean intake of energy, fat, sucrose ‐ Decrease in % children consuming high‐fat foods (90.4 to 82.2% *p* = 0.038) ‐ No significant difference in mean energy contribution of high‐fat, high‐sugar food groups	Selection bias: Low Intervention/performance bias: N/A Detection bias: High Attrition bias: Low Reporting bias: High
	Paradis 2005^25^ Canada	Changes in BMI, skin fold thickness, dietary intake, use of gym, watching television, physical activity	8 years for before and after 2 years for comparison arm	Before and after arm: ‐ Increasing trend of mean BMI (18.86–19.46 kg/m^2^) ‐ Initial improvements in physical activity/fitness returned to baseline after 8 years ‐ Decreases in key high‐sugar and high‐fat food items (65%–70% reduction in risk of consumption). However, fruit and vegetable consumption also decreased. Comparison arm: ‐ Intervention group had slower increase in skin fold thickness but no difference in BMI: intervention mean BMI 17.24–19.04 kg/m^2^, comparison 17.76–19.8 kg/m^2^ ‐ Comparison group had increased frequency of gym class and improved run/walk test results compared to intervention group ‐ No difference between groups in watching television and diet	Selection bias: Low Intervention/performance bias: N/A Detection bias: Low Attrition bias: Low Reporting bias: Low
	Adams 2005^26^ Canada	Changes in BMI, self‐reported dietary habits, physical activity and watching television	4 years	‐ No significant change in BMI (20.20 ± 5.13 to 20.27 ± 4.37 kg/m^2^, *p* = 0.96) ‐ Significant increase in self‐reported physical activity ≥30 min/day (71%–94%, *p* < 0.001) ‐ Significant decrease in television watching television ≤2 h/day (36%–63%, *p* < 0.001) ‐ Nonsignificant trend toward higher diet quality (52%–55%, *p* = 0.58)	Selection bias: Low Intervention/performance bias: N/A Detection bias: High Attrition bias: Low Reporting bias: High
The Sandy Lake Health and Diabetes Project: The Sandy Lake School‐Based Diabetes Curriculum (SLHDP)	Saksvig 2005^27^ Canada	Changes in health knowledge, % body fat, BMI, dietary intake	1 year	‐ Mean BMI increased 20.5–21.5 kg/m^2^, and body fat % from 29.8%–31% (*p* < 0.001). ‐ Energy from total fat decreased 33.8% to 31.9% (*p* < 0.05) ‐ Increase in knowledge of foods low in fat, overall health knowledge, dietary self‐efficacy and meeting recommended dietary fibre intake.	Selection bias: Low Intervention/performance bias: N/A Detection bias: Low Attrition bias: Low Reporting bias: Low
The Sandy Lake Health and Diabetes Project: The Sandy Lake School‐Based Diabetes Curriculum Round 2 (SLHDP2)	Kakekagumick 2013^28^ Canada	Changes in health knowledge, % body fat, BMI, dietary intake, physical activity, VO_2_ max, screen time	1.5 years	‐ Increase in dietary self‐efficacy (*p* = 0.001), health knowledge (*p* < 0.001), self‐reported screen time (*p* < 0.05) ‐ Energy derived from sugar decreased 30%–25% over 1 year (*p*‐value not reported). ‐ Increases in BMI *z*‐score (mean 1–1.3), waist circumference and body fat% (*p* < 0.001) ‐ Decreased VO_2_ max (*p* < 0.001)	Selection bias: Low Intervention/performance bias: N/A Detection bias: Low Attrition bias: Low Reporting bias: High
Good Start Program (GSP)	Mihrshahi 2017^29^ Australia	Changes in health knowledge, dietary intake, reported physical activity	1 year	‐ Improvement in knowledge and attitudes of healthy diet and physical activity ‐ No change in overall reported physical activity or discretionary foods intake ‐ Increase from 15%–27% of students reporting they ate recommended servings of vegetables (*p* < 0.0001)	Selection bias: Low Intervention/performance bias: N/A Detection bias: Low Attrition bias: High Reporting bias: Low
Together on Diabetes (TOD)	Kenney 2016^30^ USA	Changes in health knowledge, BMI, waist circumference, dietary intake, physical activity, blood A1c score, quality of life score, depression score	1 year	‐ Increased quality of life scores (75.25–83.43, *p* < 0.001) ‐ Decreased depression on screening (17.3%–9.2%, *p* < 0.001) ‐ Increased knowledge scores on diabetes prevention (11.7–14.44, *p* < 0.001) ‐ Increased self‐reported proportion of participants performing >30 min of rigorous physical activity for >1 day (32%–49%) ‐ No change in fats and sweets consumption and reduced fruit and grain consumption ‐ Decreased BMI z‐scores (2.19 vs. 2.16, *p* = 0.004), waist circumference remained stable ‐ Decreased hypertension (32.6% vs. 24.2%, *p* = 0.026) ‐ No difference in blood A1c scores	Selection bias: Low Intervention/performance bias: N/A Detection bias: Low Attrition bias: High Reporting bias: Low
	Ducharme‐Smith 2021^31^ USA	Changes in dietary intake and quality, systolic and diastolic BP, HbA1c, and BMI z‐score	1 year	‐ Decreased caloric intake (2016–1670 kcal/d, *p* < 0.001) ‐ Decreased consumption of whole grains (17.4–13.6 g/d, *p* < 0.001), n3 fatty acid (1537–1211 mg/d, *p* < 0.001), sodium (3675–3025 mg/d, *p* < 0.001), fruit (0.83–0.69 servings/d, *p* = 0.017), sugar‐sweetened beverages (1.58–1.34 servings/d, *p* = 0.032), nuts/legumes (0.97–0.75 servings/d, *p* = 0.034), red processed meat (1.94–1.58 servings/d, *p* = 0.008) ‐ No change in consumption of fat, protein, carbohydrates, vegetables, and alcohol ‐ An increased consumption of trans fat (1.3%–1.4 % energy, *p* = 0.016) ‐ No changes in diet quality ‐ Decreased systolic BP (*p* = 0.036) for participants in the highest AHEI‐2010 quartile group (highest adherence to recommended dietary components) ‐ Decreased HbA1c (*p* = 0.023) for participants in the highest AHEI‐2010 quartile group (highest adherence to recommended dietary components) ‐ No changes in BMI *z*‐score and diastolic BP	Selection bias: Low Intervention/performance bias: N/A Detection bias: Low Attrition bias: High Reporting bias: Low
	Chambers 2021^32^ USA	Changes in responsibility taking	1 year	‐ Increased responsibility of adolescents in ‘remembering to go to youth's clinic appointment’ (6.85%–11.16%) ‐ Increased responsibility of adolescents in ‘keeping track of youth's weight changes’ (37.5%–50%) ‐ Increased responsibility of adolescents in ‘deciding what youth will eat’ (60.8 to 68.84%) ‐ Increased responsibility of adolescents in ‘explaining absence from school to youth's teachers’ (22.27 to 30.95%) ‐ No change in responsibility of adolescents in deciding if and when the youth will be physically active (64.26%–64.45%)	Selection bias: Low Intervention/performance bias: N/A Detection bias: Low Attrition bias: High Reporting bias: Low
Youth Wellness Program (YWP)	Teufel‐Shone 2014^33^ USA	Changes in BMI, physical fitness measures, blood glucose	2 years	‐ Increased proportion with normal fasting blood glucose: males 46.2% vs. 34.2%, females 66.7% vs. 50.6% ‐ Increased prediabetes (fasting blood glucose 100–125 mg/dL): males 44.7 vs. 51.9%, females 24.8 vs. 31.1% (all *p* = 0.01) ‐ Improved body core and upper‐body fitness (measured by curl‐ups and push‐ups). E.g., females could perform mean 44.9 push‐ups on follow up compared to mean 32.4 at baseline (*p* < 0.001) ‐ Increasing trend in BMI (not significant)	Selection bias: Low Intervention/performance bias: N/A Detection bias: Low Attrition bias: Low Reporting bias: Low
BRAID‐Kids project (BKP)	Toth 2012^34^ Canada	Changes in weight, blood pressure, VO_2_ max, dietary intake, physical activity, screen time, blood glucose	1 year	‐ Overall physical activity decreased significantly. Fitness scores significantly improved (VO_2_ max measured, however numerical data not available) ‐ Weekend television viewing increased on Saturday mornings (2.6–3.1 h *p* = 0.025) ‐ Fruit and vegetable consumption decreased (*p* = 0.019) ‐ No significant change in high‐sugar and high‐fat food consumption. ‐ No differences in glucose, weight, waist or BP	Selection bias: High Intervention/performance bias: N/A Detection bias: Low Attrition bias: High Reporting bias: Low

Abbreviations: BMI, body mass index; BP, blood pressure; *p*, *p*‐value; VO2 max, maximum rate of oxygen consumption.

### 3.2 Intervention program characteristics

All programs were undertaken in regional and remote areas, with none in urban centres. All programs in the United States and Canada were implemented in First Nations communities.^21^, ^22^, ^24^, ^25^, ^26^, ^27^, ^28^, ^30^, ^31^, ^32^, ^33^, ^34^ In Australia, the Many Rivers Diabetes Prevention Project (MRDPP)^23^ and the Good Start Program (GSP)^29^ focused on schools with a high proportion of First Nations students. Most programs were primarily school‐based; the only exception was the Together on Diabetes (TOD)^30^, ^31^, ^32^ program, which was family‐ and home‐based. Four of the school‐based programs incorporated community aspects (MRDPP, KSDPP, SLHDP/2 and the BRAID‐Kids Project BKP). The duration of intervention ranged from 12 months (TOD) to 8 years (KSDPP), with follow‐ups performed simultaneously.

The intervention programs employed diverse strategies to address T2D (Table 1). School‐based components generally involved diabetes education incorporated into the school curriculum, which addressed healthy lifestyle behaviours and diabetes prevention. School‐based components also prioritized physical activity, established healthier food environments such as the prohibition of ‘unhealthy’ foods and provided education for family members and teachers. The TOD program utilized a family health coach to provide nutrition and exercise goals for participants at home.^30^, ^31^, ^32^ All programs sought to be culturally sensitive by involving the local First Nations community in co‐designing the intervention. Traditional components were integrated into physical games and food programs, while efforts were made to enhance workforce capacity through training community health workers and volunteers.

### 3.3 Specific study outcomes

Specific study outcomes are summarized in Table 2. They are presented by the following categories: diabetes knowledge, diet and nutrition, physical activity, anthropometry and physiology, glycemic indicators and psychosocial indicators.

#### 3.3.1 Diabetes knowledge

Six studies, evaluating four intervention programs (MRDPP, SLHDP/2, GSP and TOD) measured knowledge of diabetes through pre‐ and post‐questionnaires completed by participants and their carers.^23^, ^27^, ^28^, ^29^, ^30^, ^32^ The SLHDP/2, GSP and TOD programs reported improvements in knowledge,^27^, ^28^, ^29^, ^30^ while the MRDPP study reported an increasing trend in the proportion of boys correctly answering questions on diabetes knowledge.^23^ One study (TOD program) reported an increase in adolescents with T2D taking responsibility for their health, becoming more accountable for their own health‐related behaviours and decisions, particularly in managing diabetes risk.^32^ This included tasks such as attending clinic appointments, monitoring weight changes, making dietary decisions, explaining absences to school teachers and determining when to be physically active.^32^

#### 3.3.2 Diet and nutrition

Twelve studies measured dietary outcomes from seven programs (ZDPP, MRDPP, KSDPP, SLHDP/2, GSP, TOD and BKP).^21^, ^22^, ^23^, ^24^, ^25^, ^26^, ^27^, ^28^, ^29^, ^30^, ^31^, ^34^ Dietary outcomes assessed by questionnaires included self‐reported intake of high‐sugar foods and drinks, high‐fat foods, ‘unhealthy’ foods, vegetables and fruits. Seven studies reported reduced high sugar food and fat consumption at follow‐up compared to baseline,^21^, ^22^, ^23^, ^25^, ^27^, ^28^, ^31^ while three studies reported no change in consumption of fat and sweets.^24^, ^30^, ^34^ Results for consumption of fats, fruits and vegetables were inconsistent across studies; on follow‐up, GSP participants reported increased vegetable consumption,^29^ while fruit and vegetable consumption decreased in KSDPP, TOD and BKP.^25^, ^30^, ^34^ One study reported a nonsignificant trend toward higher diet quality at follow‐up.^26^

#### 3.3.3 Physical activity

Ten studies across eight programs (ZDPP, MRDPP, KSDPP, SLHDP2, GSP, TOD, YWP and BKP) measured physical activity and fitness outcomes.^21^, ^22^, ^23^, ^25^, ^26^, ^28^, ^29^, ^30^, ^33^, ^34^ These involved utilizing fitness centres, daily physical activity, screen time management, physical condition and participation in sports. These outcomes were assessed using a variety of methods, including self‐reported questionnaires, measurements of pulse rates and repetition capacity for specific exercises (e.g., count of curl‐ups). Six studies reported an improvement at follow‐up, including increased use of the wellness facility and lower sitting pulse rates (ZDPP),^21^, ^22^ increased self‐reported daily physical activity (KSDPP and TOD)^25^, ^26^, ^30^ and improved body core and upper‐body fitness (Youth Wellness Program, YWP).^33^ One study reported inconsistent physical activity and fitness results for BKP,^34^ while MRDPP reported no change in physical activity measures.^23^ Two studies assessed VO_2_ max values post exercise sessions with mixed results at follow‐up; SLHDP2 reported decreased VO_2_ max value,^28^ while BKP reported increased VO_2_ max value.^34^ Higher VO_2_ max values are associated with lower cardiovascular risk in young adults, being a measure of the maximum amount of oxygen that an individual uses during exercise.^35^

#### 3.3.4 Anthropometry and physiology

Eleven studies measured anthropometric and physiological outcomes for seven programs (ZDPP, MRDPP, KSDPP, SLHDP/2, TOD, YWP and BKP).^21^, ^22^, ^23^, ^25^, ^26^, ^27^, ^28^, ^30^, ^31^, ^33^, ^34^ Anthropometric outcomes included weight, BMI, skin fold thicknesses and body fat percentage, while physiological outcomes included blood pressure measurements. Only one study recorded improvement in BMI *z*‐score at follow‐up (2.19–2.16, *p* = 0.004) and decreased percentage of participants with hypertension (32.6%–24.2%, *p* = 0.026).^30^ Additionally, one study reported a downward trend in BMI.^21^ Other studies reported no change in anthropometric outcomes,^22^, ^23^, ^26^, ^31^, ^34^ while absolute mean BMI increased in SLHDP/2, KSDPP and YWP.^25^, ^27^, ^28^, ^33^ The BKP program reported no differences in weight, waist or blood pressure measurements.^34^

#### 3.3.5 Glycemic indicators

Six studies measured glycemic indicators from four programs (ZDPP, TOD, YWP and BKP).^21^, ^22^, ^30^, ^31^, ^33^, ^34^ These included fasting glucose and insulin levels, oral glucose tolerance testing, and HbA1c. Three studies demonstrated improvement in HbA1c, with a downward trend in insulin levels.^21^, ^22^, ^31^ The YWP program reported a higher number of children with normal fasting blood glucose at follow‐up but also identified a greater number of children as having pre‐diabetes.^33^ Two studies did not report any change in mean HbA1c or glucose levels.^30^, ^34^

#### 3.3.6 Psychosocial indicators

Only one study measured quality of life and mental health outcomes.^30^ Quality of life was assessed through a Paediatric Quality of Life questionnaire (a scale out of 100 points) and depression was assessed through a Patient Health Questionnaire (n%). Quality of life significantly increased at follow‐up (75.24–83.43, *p* < 0.001), while depression significantly decreased (17.3%–9.2%, *p* < 0.001).^30^

### 3.4 Risk of bias and quality assessment

Appraisal for bias was often affected by insufficient detail reported in the studies. Selection bias may have affected study findings, as participants who completed evaluations may have been more motivated than those lost to follow‐up. Most of the positive significant improvements were self‐reported data from questionnaires and may have been influenced by reporting bias. Only one study included a separate comparison group, which received their own intervention.^25^ None of the studies included a control group, potentially leading to confounding factors influencing the observed variations in outcomes. Additionally, two study evaluations were not published in peer‐reviewed journals.^23^, ^34^

## 4 DISCUSSION

This systematic review identified 14 studies evaluating 9 intervention programmes to prevent T2D in First Nations children and young people. Findings across studies were mixed. Knowledge of diabetes and healthy lifestyle habits consistently improved across studies; however, these improvements did not always translate into improvements in other health outcomes. The programmes identified were predominantly school‐based, with 8 of the 9 programs in this review being implemented in a school setting. A key strength of these programs was their co‐design and participatory action research approach involving First Nations stakeholders. This ensured that programs addressed priority areas identified by the community.^36^

Five studies reported a decrease in the consumption of high‐sugar food and drinks, with only one study demonstrating an improvement in BMI *z*‐score and three studies reporting improvements in HbA1c. It is possible that the interventions prevented the worsening of indicators, but this could not be demonstrated due to the lack of a control group. For the study that assessed mental health outcomes, quality of life increased at follow‐up, while depression scores decreased. These mixed findings could be attributed, in part, to the diverse study populations involved. These populations consisted of First Nations children and young adults residing in various geographical locations (Canada, the United States and Australia). Additionally, variations in program implementation, duration, and delivery, as well as the tools and methods used to measure outcomes, study design and methodology may have contributed to the inconsistent results. Furthermore, systemic inequities that continue to impact First Nations populations, particularly in rural and remote communities, cannot be overlooked.^37^

The unique challenges encountered by the First Nations communities can vary considerably across different settings, which may subsequently influence the outcomes observed in research studies. As such, it is essential to adapt interventions to the specific local context to ensure their effectiveness.^38^ While adaptation is necessary, this may introduce complexities when assessing program effectiveness and expanding or transferring them to different settings. The programs in this review did not address the complex social and economic challenges experienced by First Nations communities. These include food security, housing, education and access to healthcare, all of which have been found to limit the capacity for healthy behaviour change.^39^, ^40^ Social determinants of health play a key role in improving health outcomes.^13^ Future intervention programs need to address social determinants of health in First Nations communities in addition to broader health outcomes.

There is a clear preference for school‐based strategies, consistent with previous literature that has emphasized school‐based interventions in obesity prevention in First Nations children and young people.^41^, ^42^, ^43^, ^44^, ^45^, ^46^ School‐based programs create a supportive environment that promotes healthy behaviours and empowers children and young people to make positive choices for their health.^47^ However, the school setting has potential limitations, particularly for First Nations populations in regional and remote areas where family and communities may play a more significant role in influencing a child's health outcomes, and school attendance is reduced.^48^ Although programs employed components such as information sessions for parents or community events, strategies focusing on home and community were not consistently emphasized across the school‐based interventions. It is important to note that the TOD program, the only home‐based intervention in our review, reported the most significant improvements in most of its measured outcomes. A family‐based program on obesity prevention in the United States focused on diet, exercise, and behaviour modification demonstrated significant improvements in health outcomes, including reduced BMI z‐score and insulin resistance, a year after the intervention was implemented in an ethnically diverse paediatric population.^49^ In Australia, a community‐based obesity program for both children and parents also demonstrated significant improvements in BMI *z*‐score, diet and physical activity; however, there was less retention amongst Aboriginal children.^50^ This supports the need for further research into prevention strategies which are led by and effective in First Nations family units and communities.

Prioritizing cultural relevance and actively involving First Nations communities throughout the process has also been essential characteristics of programs identified in prior systematic reviews focusing on childhood nutrition and obesity prevention in First Nations populations in Canada and the United States.^41^, ^42^, ^43^, ^44^, ^45^, ^46^, ^51^ Our review adds to this previous research by highlighting the importance of engaging with members of First Nations communities during the development, implementation and evaluation phases. This was vital for fostering community participation, ownership and program sustainability, and being relevant to the impact on quality of life and mental health. Developing a program that incorporates perspectives from all stakeholders enables a more thorough evaluation of the challenges and possibilities for program implementation.^46^ Within First Nations communities, this approach allows for the integration of traditional healing practices, cultural values and community norms into the intervention, potentially increasing its acceptability and effectiveness within the community. By addressing the unique needs and strengths of First Nations communities and involving them actively in the process, interventions can promote a sense of cultural pride and build resilience, ultimately working toward improving overall health outcomes.^48^

The findings suggest that culturally adapted interventions, which prioritize cultural relevance and actively involve First Nations communities, are essential in T2D prevention strategies for First Nations children and young people. Aside from school‐based approaches, this review suggests the need for multi‐faceted community and home‐based interventions. Involving families in these interventions may create a supportive environment that encourages healthy behaviours, as family dynamics play a critical role in the lifestyle choices of young people.^52^ Research has shown that integrating culturally relevant education and support systems into home‐based programs can effectively empower First Nations youth to take charge of their health, ultimately reducing the risk of T2D and its associated complications.^53^ These interventions should also incorporate mental health and quality of life components, as emerging evidence suggests that mental health and well‐being are associated with the risk of T2D and long‐term complications upon diagnosis of T2D among First Nations young people.^54^, ^55^ Interventions should also be tailored to this specific age group, due to a more rapid disease progression and a distinct metabolic profile.^2^, ^3^, ^4^, ^5^ Additionally, traditional models of care often fail to resonate with younger populations, who may benefit more from innovative, peer‐led and community‐based approaches that foster engagement and motivation. Peer education models can enhance the effectiveness of diabetes prevention programs by leveraging social connections and relatable experiences among youth.^56^ This review also highlights the lack of evidence among young adults, compared to younger youth, suggesting a need for effective interventions targeting this age group.

### 4.1 Limitations

Limited studies led to a small number of included studies in the results. A substantial number of studies (*n* = 18) were excluded due to having follow‐up periods of <12 months, demonstrating the challenges in sustaining programs and conducting long‐term evaluations. Studies that did not provide age or ethnicity‐specific data on children and young people were also excluded. Furthermore, studies were excluded if they did not explicitly address the prevention of T2D in First Nations children and young people. Although our review sought to include children and young people, only participants of school age were included as there was no specific data on young adults (18–25 years of age) reported. Additionally, as these intervention programs were only implemented in rural and remote settings among specific First Nations populations, the findings may not be generalizable across different settings, such as urban areas or First Nations communities outside Australia, Canada and the United States. The absence of studies representing these populations also reveals an important research gap.

## 5 CONCLUSION

Our findings highlight the importance of culturally adapted interventions, participatory research approaches and family and home‐based programmes in preventing T2D among First Nations children and young adults. Understanding the multifaceted nature of these interventions and the diverse contexts in which they are implemented is crucial for developing effective and sustainable programmes. These interventions should include long‐term follow‐up to assess the sustained impact of interventions and explore how changing contexts influence outcomes. Addressing systemic inequities, such as food security, discrimination, housing, education and access to healthcare, is critical. Research should also explore integrating traditional knowledge with modern practices and innovative tools such as culturally tailored digital health solutions. Further research and collaboration with First Nations communities are essential to ensure that interventions are truly impactful and resonate with the unique cultural and environmental contexts of First Nations children and young adults worldwide.

## AUTHOR CONTRIBUTIONS

M. C., J. N., R. H. N., D. M. and A. T. were responsible for the study design. Data collection was completed by M. C., J. N. and R. H. N. M. C., J. N., R. H. N., D. M. and A. T. interpreted the results. M. C. and J. N. drafted the initial version of the manuscript, which was critically reviewed, and the final version was approved by all co‐authors. Each of the authors confirms that this research has not appeared in a published abstract nor been posted on a preprint server.

## FUNDING INFORMATION

Australian Government Department of Health and Aged Care.

## CONFLICT OF INTEREST STATEMENT

The authors have no conflicts of interest to disclose.

## Supporting information


**Appendix A.** Example keyword search strategy according to the PICO.
