# Supplementary material for: A systematic review of prevention strategies for type 2 diabetes in First Nations children and young people
Source: Pediatr Obes. 2025 Mar 10;20(6):e70009. doi: 10.1111/ijpo.70009 (PMC12056536; doi:10.1111/ijpo.70009)
Supplement: Supplementary file 1 — Appendix A. Example keyword search strategy according to the PICO. [file IJPO-20-e70009-s001.docx]

**Appendix A** Example keyword search strategy according to the PICO

Included search terms were: (paediatrics or children or child or young person) OR (young people or youth or adolescents or young adults) OR (children or adolescents or youth or child or teenager) AND (indigenous or native or aboriginal or indians or first nations) OR (aboriginal and torres strait islander or indigenous Australians) OR (aboriginal or indigenous or first nations or native or Indian) OR (inuit or eskimos or inuk) OR (native american or american indian or indigenous or native tribes or native people) OR (oceanic ancestry group) OR (maori) AND (diabetes type 2 or diabetes mellitus type 2 or diabetes 2) OR (obesity or overweight or fat or obese or unhealthy weight or high bmi) OR (glucose intolerance) OR (impaired glucose tolerance) OR (adiposity or obesity or overweight) OR (metabolic syndrome or metabolic disease) OR (insulin resistance) AND (prevention) OR (prevent)
